# Small interfering RNA-producing loci in the ancient parasitic eukaryote *Trypanosoma brucei*

**DOI:** 10.1186/1471-2164-13-427

**Published:** 2012-08-27

**Authors:** Christian Tschudi, Huafang Shi, Joseph B Franklin, Elisabetta Ullu

**Affiliations:** 1 Department of Epidemiology of Microbial Diseases, School of Public Health, Yale University, New Haven, CT, 06536, USA; 2 Department of Internal Medicine, School of Medicine, Yale University, New Haven, CT, 06536, USA; 3 Department of Cell Biology, School of Medicine, Yale University, New Haven, CT, 06536, USA

**Keywords:** Argonaute, Trypanosome, Dicer-deficient, Retrotransposon, Inverted repeat, Convergent transcription unit, siRNA

## Abstract

**Background:**

At the core of the RNA interference (RNAi) pathway in *Trypanosoma brucei* is a single Argonaute protein, *Tb*AGO1, with an established role in controlling retroposon and repeat transcripts. Recent evidence from higher eukaryotes suggests that a variety of genomic sequences with the potential to produce double-stranded RNA are sources for small interfering RNAs (siRNAs).

**Results:**

To test whether such endogenous siRNAs are present in *T. brucei* and to probe the individual role of the two Dicer-like enzymes, we affinity purified *Tb*AGO1 from wild-type procyclic trypanosomes, as well as from cells deficient in the cytoplasmic (*Tb*DCL1) or nuclear (*Tb*DCL2) Dicer, and subjected the bound RNAs to Illumina high-throughput sequencing. In wild-type cells the majority of reads originated from two classes of retroposons. We also considerably expanded the repertoire of trypanosome siRNAs to encompass a family of 147-bp satellite-like repeats, many of the regions where RNA polymerase II transcription converges, large inverted repeats and two pseudogenes. Production of these newly described siRNAs is strictly dependent on the nuclear DCL2. Notably, our data indicate that putative centromeric regions, excluding the CIR147 repeats, are not a significant source for endogenous siRNAs.

**Conclusions:**

Our data suggest that endogenous RNAi targets may be as evolutionarily old as the mechanism itself.

## Background

The “natural” or endogenous RNA interference (RNAi) pathway functions as a genome immune defense mechanism to maintain genome integrity, prevents viral infection and limits the potential deleterious consequences of transposon/retroposon mobilization [1-3]. Whereas deep sequencing of endogenous small interfering RNAs (siRNAs) has confirmed that the large majority of siRNAs in several model organisms, including insects [4], plants [5] and mammals [6,7], are indeed derived from retroposons and transposons, these studies also uncovered new classes of small RNAs originating among others from regions of the genome, where convergent transcription occurs or from loci predicted to generate double-stranded RNA (dsRNA) by a fold back mechanism, i.e. inverted repeats. In addition, tRNA- and snoRNA-derived RNA fragments have recently been added to the catalogue of small RNAs implicated in RNAi-related gene silencing pathways [8-12].

The processing of dsRNA is executed by a member of the Dicer family of RNase III-related enzymes to yield a variety of 21–30 nt small dsRNAs that are then loaded into a specific class of Argonaute (AGO)-family proteins [13,14]. RNAi was first described in the parasitic protozoan *Trypanosoma brucei* in 1998 [15] and to date we know that there are two distinct Dicer-like enzymes in these organisms, namely *Tb*DCL1 [16] and *Tb*DCL2 [17], which are mostly present in the cytoplasm and in the nucleus, respectively. siRNAs generated by both Dicers are transferred to *Tb*AGO1, the sole slicer responsible for cleavage of target transcripts [18,19] with the assistance of *Tb*RIF4, a 3’-5’ exonuclease [20].

Soon after the discovery of RNAi in *T. brucei*, “old-fashioned” sequencing of an 20–30 nt RNA library revealed that the two main classes of retroposons, namely ingi and SLACS, were sources of siRNAs [21] and subsequent sequencing of siRNAs derived from *Tb*AGO1 immunoprecipitates uncovered a new class of siRNAs from 147-bp tandem units [17], which we named CIR147 repeats (Chromosome Internal Repeats). These satellite-like repeats are located in non-telomeric regions of *T. brucei* chromosomes 4, 5, and 8 and were independently identified as part of putative centromeric regions [22], although at present we cannot exclude the possibility that these repeats also exist on the other chromosomes. In addition, functional studies were consistent with a major role for endogenous RNAi in *T. brucei* to maintain genome integrity [17,23]. On the other hand, the unique organization of the trypanosome genome into long directional gene clusters with many sites of convergent transcription [24] raised the possibility of additional sources of siRNAs and thus may be pointing to new role(s) for RNAi in these organisms. Thus, using deep sequencing we surveyed the small RNAs associated with Argonaute 1 in wild-type cells, as well as in cells devoid of either one of the two Dicers to gauge their respective role in small RNA production.

## Results

### Small RNA data sets

To provide a comprehensive catalogue of small RNA-generating loci in the procyclic *T. brucei* YTat 1.1 strain [25], we subjected RNAs associated with *Tb*AGO1 to next-generation sequencing on the Illumina platform and the distribution of siRNAs along the *T. brucei* 11 mega chromosomes (GeneDB version 5) was analyzed using a bioinformatics pipeline described in Methods. In addition to wild-type cells, we surveyed small RNAs in cells lacking either *Tb*DCL1 or *Tb*DCL2, to gain further insight into the distinct role of the two Dicers in the RNAi pathway. Alignment to the reference *T. brucei brucei* TREU 927 genome [24] with 93% identity yielded 8,775,792, 7,821,630 and 4,722,271 total reads (Table 1), representing 842,296, 744,681 and 545,597 unique sequences (Additional file 1) for the wild-type, *dcl1*^*−/−*^ and *dcl2*^*−/−*^ libraries, respectively (The sequence data from this study have been submitted to the NCBI Sequence Read Archive - SRA at http://www.ncbi.nlm.nih.gov/Traces/sra/sra.cgi - under accession no. SRA057341). The overall distribution of reads did not change noticeably, if the alignment was performed by increasing the % identity to 95% or 100%, albeit the read density was reduced uniformly.

**Table 1:** Summary of total small RNA reads

	**wt**	***dcl1***^***-/-***^	***dcl2***^***-/-***^
Total reads	10,001,135	9,199,808	5,829,677
Reads mapped to the			
11 mega chromosomes	8,775,792	7,821,630	4,722,271
Annotated reads	6,826,892	5,987,935	3,400,947
Unassinged reads	1,948,900	1,833,695	1,321,324
Unmapped reads	1,225,343	1,378,178	1,107,406

To categorize small RNA-producing loci, we inspected the aligned data for regions that corresponded to annotated features. In addition, we surveyed the *T. brucei* genome for tandem and inverted repeats, which to our knowledge has not been done systematically, and asked whether they are sources of small RNAs. This strategy allowed us to annotate 6,826,892 of the reads (78%) in the wild-type library and establish seven categories of *bona fide* siRNA sources (Table 2), which were analyzed further as described below. The reads not matching to the 11 mega chromosomes (1,225,343 or 12%) might arise from unsequenced regions of the genome or from sequencing errors. Indeed, an additional 638,046 reads aligned to the *T*. *brucei gambiense* genome, a closely related trypanosomatid, with no genomic regions rising above background levels (data not shown). In addition, *de novo* assembly of the remaining reads did not generate contigs that would point to additional genuine siRNA-producing loci (data not shown).

**Table 2:** **Summary of small RNA classes in procyclic* T. brucei***

	**wt**	***dcl1***^***-/-***^	***dcl2***^***-/-***^
ingi/RHS	3,555,719 (52.1%)	3,151,779 (52.6%)	2,380,772 (70.0%)
SLACS	1,793,319 (26.3%)	1,546,465 (25.8%)	486,558 (14.3%)
CIR147	586,988 (8.6%)	339,312 (5.7%)	4,953 (0.2%)
IR^a^	452,056 (6.6%)	282,956 (4.7%)	105,792 (3.1%)
CTU^b^	179,694 (2.6%)	265,799 (4.4%)	151,045 (4.4%)
Pseudogenes	55,460 (0.8%)	45,079 (0.8%)	179 (0.01%)
Miscellaneous^c^	92,099 (1.4%)	70,815 (1.2%)	4,169 (0.11%)
rRNAs	97,847 (1.4%)	265,895 (4.4%)	253,779 (7.5%)
t/snoRNAs^d^	9,327 (0.1%)	18,574 (0.3%)	13,272 (0.4%)

^a^IR, long inverted repeats (see Table 3).

^b^CTU, convergent transcription unit, see Additional File 2 for details.

^c^Miscellaneous, see Additional File 3 for details.

^d^t/snoRNAs, tRNAs, snRNAs and snoRNAs.

**Figure 1:**
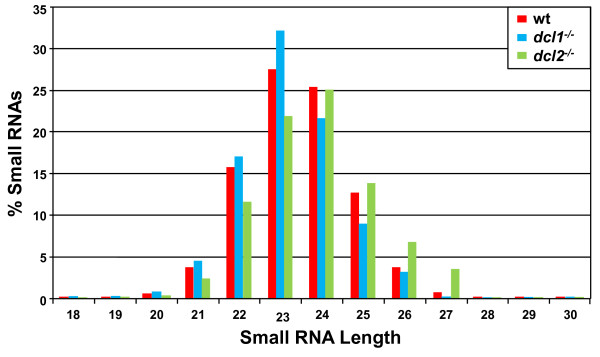
**Size distribution of analyzed* T. brucei *small RNAs in wild-type (wt),* dcl1***^***−/−***^**and* dcl2***^***−/−***^**cells.**

The small RNAs recognized as siRNAs displayed a distinct size distribution ranging in size from 22 to 25 nucleotides (Figure 1, wild-type), which mirrors our previous estimates [17,21,23]. siRNAs isolated from cells deficient in *Tb*DCL2 were slightly larger (Figure 1 and see below), in line with our previous observation that siRNAs processed in an *in vitro* extract from *dcl2*^*−/−*^ cells were one nucleotide longer than the wild-type size [17]. In contrast, reads aligning to rRNAs (97,847 total reads or 1.4%) and tRNAs/snoRNAs (9,327 total reads or 0.1%), did not display a length distribution characteristic of siRNAs, but had a broad distribution between 18 and 32 nucleotides (data not shown). Furthermore, we carefully inspected reads originating from tRNAs and snoRNAs, since recent reports have highlighted a novel class of small RNAs originating from these structural RNAs [8-12]. Our analysis revealed that reads aligning to trypanosome tRNAs and snoRNAs had a random distribution and did not suggest cleavage in a specific manner, reminiscent of tRNA- or snoRNA-derived small RNAs implicated in RNA silencing, Thus we classified them as degradation products. Finally, we did not find evidence for the presence of micro RNAs in the libraries we generated from the procyclic *T. brucei* YTat 1.1 strain.

### The retroposons ingi and SLACS are major sources for small RNAs

**Figure 2:**
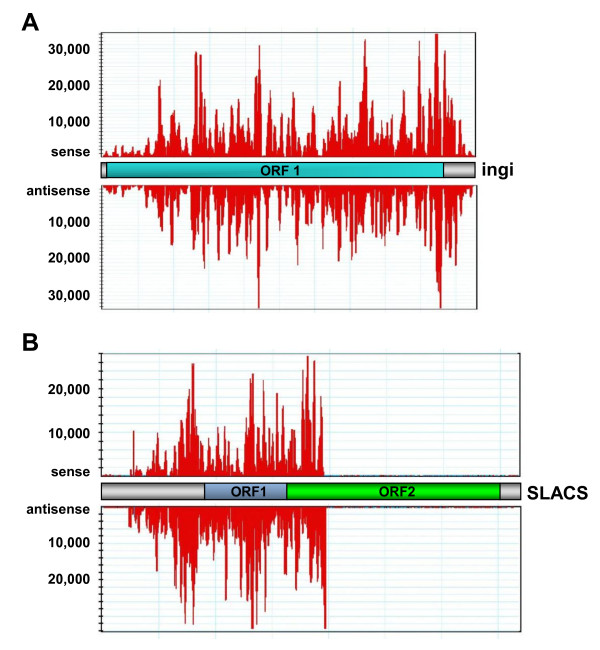
**Distribution of small RNAs aligning to retroposon elements. (A)** ingi is a 5.25 kb element composed of an ingi-specific 4.75 kb fragment flanked by two separate halves of the RIME element family [28,29]. This element contains a single ORF (ORF1) flanked by two RIME elements. 2,326,864 reads in the wild-type library aligned to this element with 1,1321,119 reads from the sense and 1,194,745 reads from the antisense strand. Note that sense and antisense small RNA reads are shown separately. **(B)** The SLACS element is 6.8 kb long with two predicted ORFs. Sense (640,935 reads) and antisense (1,152,384 reads) small RNA reads are shown separately.

Consistent with our earlier results [21], the retroposons ingi and SLACS were by far the two major sources of small RNAs accounting for 78% of all reads in the wild-type library (Table 2). Ingi is part of a group of related, but rather heterogeneous retroposon-like sequences present throughout the genome, which also includes the ribosomal inserted mobile elements (RIME) and the retrotransposon hot spot (RHS) family [26,27]. In our analysis we pooled all the reads aligning to these various elements and thus in the wild-type library this group of retroposons was responsible for 52% of the siRNAs (Table 2). The “LINE-like” ingi is a 5.25 kb element composed of an ingi-specific 4.75 kb fragment flanked by two separate halves of the RIME element family [28,29]. A potentially functional retroposon contains a single long ORF (4,971 bp), which encodes a 1,657 amino acid protein with a predicted reverse transcriptase and endonuclease domain (Figure 2A and refs. [30,31]). We used such an element to gauge the distribution of siRNAs, which turned out to be fairly even along the entire retroposon with no obvious gaps and with a similar distribution of reads coming from the sense or antisense strand (Figure 2A).

SLACS, or Spliced Leader Associated Conserved Sequence, is a site-specific non-LTR retroposon that integrates exclusively into the spliced leader (SL) RNA gene [32]. Our wild-type Ytat 1.1 strain, which was used for the small RNA sequencing described here, has between 16 to 18 copies of the SLACS element per haploid genome (ref. [33] and data not shown). The element is 6.8 kb long and the two predicted ORFs encode a putative reverse transcriptase and endonuclease domain (Figure 2B). In the wild-type library 26% of the total reads (16% of the unique reads) aligned to this retroposon with approximately twice as many reads originating from the antisense strand. However, in contrast to ingi, siRNAs were restricted to the 5’ half of the element with very few reads in the 3’ half (Figure 2B). Consistent with our analysis of siRNA abundances in *Tb*DCL1 and *Tb*DCL2 KO cells by Northern blots [17], siRNAs numbers in cells lacking *Tb*DCL1 were slightly lower, but were reduced to 14% in *Tb*DCL2 KO cells (Table 2). The uneven distribution of siRNAs was maintained in both KO libraries.

### Small RNAs originate from CIR147 repeats, but not from other tandem repeats

In the wild-type library 8.6% of the total reads (586,988 reads) aligned to the CIR147 tandem repeats (Table 2). The abundance of CIR147 siRNAs was reduced slightly to 5.7% in the absence of *Tb*DCL1, whereas in the DCL2KO library only 0.2% of the siRNAs originated from these repeats, thus confirming our functional studies that *Tb*DCL2 has a primary role in the generation of siRNAs from CIR147 repeats [17].

CIR147 repeats on chromosomes 4, 5 and 8 were previously identified as part of putative *T. brucei* centromeric regions based on etoposide-mediated topoisomerase-II cleavage [22]. Since centromeres in the fission yeast *Schizosaccharomyces pombe* are under the control of the RNAi pathway [34], we surveyed other predicted centromeric regions in *T. brucei* for the production of small RNAs. However, none of the putative centromeric regions on chromosomes 1, 2, 3, 6 and 7 [22] showed a significant accumulation of reads as compared to flanking regions (data not shown). Of note is that putative centromeres were not mapped on the largest three chromosomes, i.e. 9, 10 and 11 [22]. The lack of reads at the putative centromeres on chromosomes 2, 3 and 7 was particularly intriguing, since they contain large arrays of tandem repeats of 29, 120 and 59 base pairs, respectively (ref. [22] and data not shown), suggesting that short tandem repeats *per se* are not destined to be a source for small RNAs in procyclic *T. brucei*. To test this hypothesis, we used the program tandem repeats finder [35], tabulated all repeat arrays on the 11 mega base chromosomes (data not shown) and then manually inspected repeats with a size >10 bp and a copy number >10 for aligned reads. Although many additional tandem repeats were identified, none was found to be a source of small RNAs (data not shown). Thus, it appeared from this analysis that the CIR147 repeats are the only tandem repeats in the *T. brucei* genome generating siRNAs and that putative centromeric regions not harboring CIR147 repeats are devoid of siRNAs.

### New loci for small RNA generation: pseudogenes and inverted repeats

**Figure 3:**
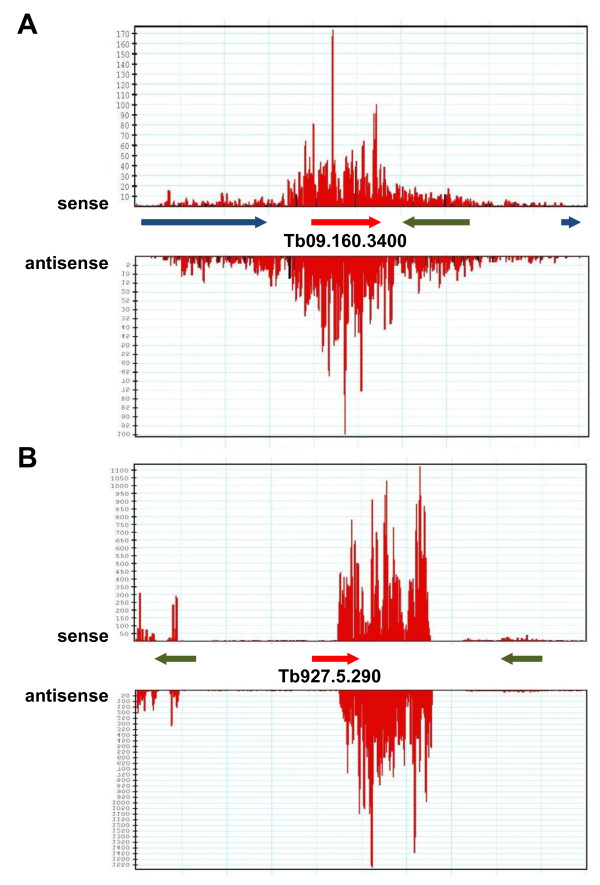
Small RNAs originating from two annotated pseudogenes, namely Tb09.160.3400 (A) and Tb927.5.290 (B). Sense and antisense reads are shown.

Once we recognized all the siRNAs originating from retroposons and CIR147 repeats, we were left with 13% (890,866 reads) of the reads aligning to potential small RNA-producing loci in the *T. brucei* genome. Based on similar studies in *Drosophila* and mammals [6,36,37], we considered additional possible sources of small RNAs, i.e. pseudogenes and inverted repeats. In the GeneDB release 5 of the *T. brucei* genome [24], which was used in our analysis, there are 1,279 annotated pseudogenes with the majority assigned to variant surface glycoprotein (VSG) genes and expression-site-associated genes (ESAG), as well as a few for other protein coding genes. In the library from wild-type procyclic cells there was no apparent increased accumulation of reads at VSG or ESAG pseudogenes, as compared to flanking regions, but there were two regions, currently annotated as pseudogenes but for which the corresponding “genes” have not yet been identified, that generated small RNAs, namely Tb927.5.290 (51,758 total reads) and Tb09.160.3400 (3,702 total reads). Small RNAs from these two loci appeared to be predominantly generated by *Tb*DCL2, since ablation of *Tb*DCL1 barely affected the accumulation of siRNAs, whereas the lack of *Tb*DCL2 reduced the small RNA levels to 5% or lower relative to wild-type (Table 2, Additional file 4 and Figure 3).

To the best of our knowledge, the current *T. brucei* genome has not been surveyed for the presence of large inverted repeats (IR). Thus, we used the program inverted repeat finder [38], as well as BLAST [39] to align each chromosome with itself, and identified 7 inverted repeats ranging in size from 422 bp to 10,011 bp (Table 3). Remarkably, at the sequence level each repeat pair is essentially identical with only 14 mismatches for the longest one. To ascertain that these were *bona fide* inverted repeats and not genome assembly artifacts, we verified the presence of IR3 (3,636 bp) and IR4 (2,520 bp) in our YTat 1.1 strain by PCR (data not shown).

**Table 3:** **Small RNAs at long inverted repeats in* T. brucei***

**Name**	**Start/End**	**RL**^a^	**Identity**	**wt**	***dcl1***	***dcl2***
		**(bp)**		**total reads**	**%wt**	**%wt**
Tb927_02_IR1	898600/902200	422	100%	403	52%	9%
Tb927_02_IR2	994300/100060	10,011	99% (14^b^)	8,681	56%	24%
Tb927_07_IR3	1125000/1134900	3,636	99% (1)	372,045	73%	41%
Tb927_07_IR4	1889100/1896500	2,520	100%	55,854	71%	103%
Tb927_08_IR5	1048400/1059500	1,743	99% (5)	1,390	108%	49%
Tb927_10_IR6	467000/478000	853	99% (8)	4,387	38%	6%
Tb927_11_IR7	4219000/4231000	1,814	99% (3)	9,296	52%	2%

^a^RL, repeat length.

^b^number of mismatches between the two repeats.

**Figure 4:**
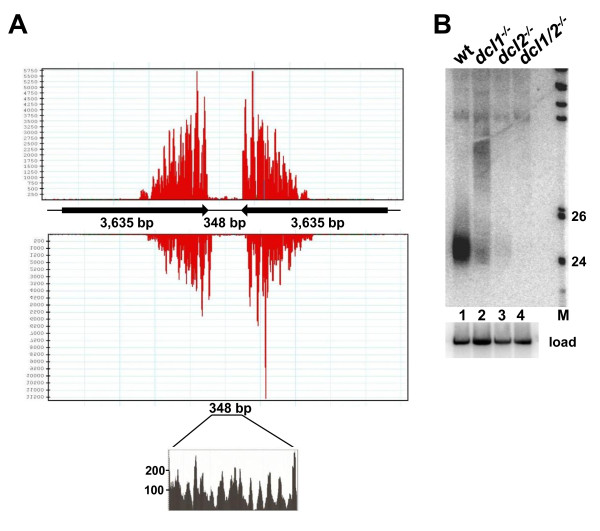
**Long inverted repeats are a source of small RNAs. (A)** Small RNA distribution on IR3. Reads were distributed randomly on the two repeats during alignment. An enlargement of the region in-between the two repeats is shown below. **(B)** Low-molecular weight RNAs isolated from various cell lines as indicated above each lane were separated by denaturing gel electrophoresis and analyzed by Northern hybridization with an IR3-specific probe. Hybridization to 5S rRNA was used as a loading control (load, bottom panel). See additional file 5 for sequences of the hybridization probes.

Inverted repeats accounted for 6.6% of small RNAs in the wild-type library, with the number of reads per inverted repeat varying from 403 for IR1 to 372,045 for IR3 (Table 3). Curiously and similar to what we noticed with the SLACS retroposon (Figure 2B), the small RNAs originated from restricted regions of the inverted repeat and did not cover the entire length (Figure 4A). In addition, the region separating the two repeats was also a source of small RNAs, suggesting that transcription occurred on both strands. In all cases but one (IR5), the read count was reduced to between 39% and 75% of wild-type levels in the absence of *Tb*DCL1. The involvement of *Tb*DCl2 in the generation of small RNAs from inverted repeats varied, with small RNAs derived from IR1, IR6 and IR7 clearly dependent on the nuclear Dicer, whereas IR2, IR3 and IR5 appeared to be less dependent (Table 3). Finally, the generation of small RNAs originating from IR4 appeared to be only slightly dependent on either Dicer by the sequencing analysis. The generation of small RNAs from inverted repeats and their dependence on the two Dicers was validated by Northern blotting for IR3 (Figure 4B).

### Convergent transcription units are a source of small RNAs

**Figure 5:**
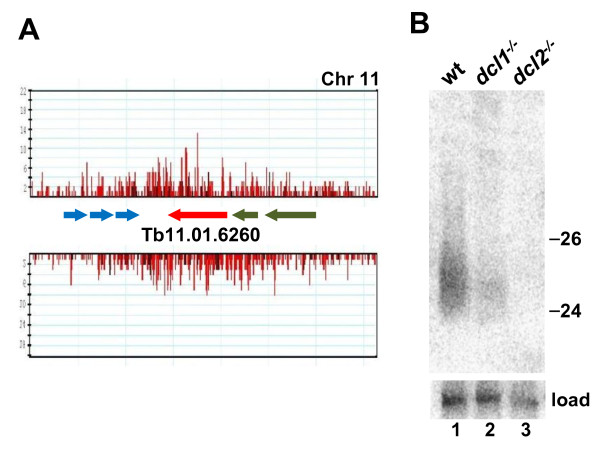
**Validation of small RNA accumulation at CTU50. (A)** Small RNA distribution. **(B)** Low-molecular weight RNAs isolated from various cell lines as indicated above each lane were separated by denaturing gel electrophoresis and analyzed by Northern hybridization with two oligonucleotides representing the most abundant reads. Loading control (load), hybridization to 5S rRNA. See additional file 5 for sequences of the hybridization probes.

One peculiar feature of the *T. brucei* genome is the organization of protein coding genes into long directional clusters [24]. A corollary of this trait is that in chromosomal regions were units converge, there is the potential for overlapping (bi-directional) transcription, which could potentially lead to the formation of dsRNA and subsequent small RNAs. We surveyed 49 convergent transcription units (CTUs) on the 11 megabase chromosomes and in each case we detected significant accumulation of reads in these regions, as compared to flanking sequences (Additional file 2). For the majority of CTUs accumulation of small RNAs was not dependent on *Tb*DCL1, except for CTU4, CTU17, CTU40 and CTU41, where ablation of *Tb*DCL1 reduced the read count to about 60% of wild-type levels. In contrast, small RNA accumulation at all CTUs was critically dependent on *Tb*DCL2. We validated the production of small RNAs at one of the convergent units (CTU50) by Northern blot analysis, which also corroborated the involvement of *Tb*Dcl2 in the formation of small RNAs (Figure 5).

**Figure 6:**
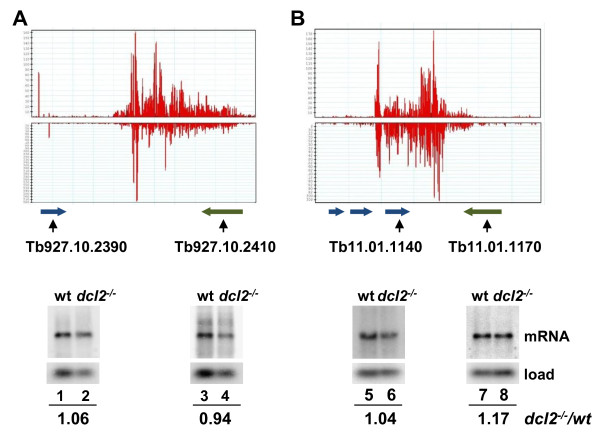
**Steady-state mRNA levels at two convergent transcription units in wild-type and DCL2KO cells. (A)** and **(B)** Total RNA was prepared from wild-type and *dcl2*^*−/−*^ cells and analyzed by Northern hybridization with a probe specific for the genes indicated below the read distribution. See additional file 3 for sequences of the hybridization probes. The hybridization was quantitated by PhosphorImager analysis, normalized to the loading control (tRNA) and expressed as a ratio between *dcl2*^*−/−*^ and wt.

The production of siRNAs at convergent transcription units raises the question whether these siRNAs modulate gene expression, since they overlap with annotated genes. We chose two CTUs (CTU36 and CTU48) and monitored the steady-state levels of two mRNAs in each unit in RNA isolated from wild-type and *dcl2*^*−/−*^ cells by Northern blot analysis (Figure 6). In both CTUs there was no detectable change in the accumulation of mRNAs in the absence of *Tb*DCL2, thus questioning the contribution to gene expression of siRNAs generated in convergent transcription units.

## Discussion

Given the wide distribution of RNAi and related phenomena in all branches of the eukaryotic lineage, a case can be made that the RNAi mechanism originated early during eukaryotic evolution and, as an extension, the repertoire of small RNAs generated by the RNAi pathway should have an evolutionary history. To explore this hypothesis, we have been studying the mechanism and biological function of RNAi in the ancient parasitic protozoan *T. brucei*. This lead to the realization several years ago that the two retroposons in the genome, namely ingi and SLACS, were a source of siRNAs, thus implicating RNAi in maintaining genome integrity [21]. The next observation we made was that a subclass of putative centromeres containing 147-bp tandem repeats were also generating siRNAs [17]. In the current study we aimed to annotate all small RNA-producing loci in insect-form derived trypanosomes by deep-sequencing RNAs associated with the sole Argonaute 1 slicer. In addition, to further our understanding of the specific role of the two Dicers in this organism, we surveyed small RNAs in cells deficient in either the nuclear or cytoplasmic Dicer.

Our results exposed a number of intriguing features and thus raise numerous questions. Firstly, siRNAs originating from SLACS were restricted to the 5’ half of the element (Figure 2B), suggesting a possible avenue for the generation of double-stranded RNA. Our studies on the expression of the SLACS element suggested that transcription initiates at the +1 position of the interrupted spliced leader RNA gene and continues through the 5’ UTR and ORF1 [33]. In addition, preliminary experiments indicated a detectable amount of antisense transcription, although the low level of transcription made it impossible to pinpoint the extent of transcription, let alone the site of transcription initiation [33]. Nevertheless, having a defined landmark from our read alignments, it might be possible in future experiments to delineate the origin of the double-stranded RNA.

Secondly, the CIR147 repeats present in putative centromeric regions on three chromosomes were the only tandem repeats in the genome giving rise to small RNAs, despite the presence of other short tandem repeats either in putative centromeres or other genomic regions. What is so special about the CIR147 repeats that they are under the control of the RNAi pathway and how are siRNAs, i.e. dsRNA, produced from these arrays? At present there is no knowledge of what constitutes a centromere in trypanosomes and whether heterochromatin is crucial for centromere function. More importantly, a very basic question to put forward is whether the silent transcriptional status of the CIR147 repeats in wild-type cells [17] is caused by a heterochromatic state of the locus. In *Drosophila* and in yeast, H2AZ was reported to be involved in heterochromatic silencing [40,41]. This histone variant has been characterized in *T. brucei* and shown to function in concert with a novel H2B variant, H2BV [42]. By ChIP, both proteins were shown to be associated with highly repetitive DNA, including the mini-chromosomal 177-bp repeats, the expression site-proximal 50-bp repeats, and telomeric repeats. Intriguingly, H2AZ and H2BV do not co-localize with sites of nascent RNA transcription, suggesting that they are primarily enriched at transcriptionally inactive regions of the genome. Given our data, it is tempting to speculate that these two histone variants might be involved in the regulation of the transcriptional status of the 147-bp repeat clusters, a possibility we are currently investigating. Taking advantage of RNAi-deficient cells, we know that both strands of the CIR147 repeats generate transcripts of over 10 kb and based on α-amanitin sensitivity are synthesized by RNA polymerase II [17]. Although the latter observation might be in line with studies in other organisms, where it has been shown that RNA polymerase II appears to play a direct role in heterochromatin assembly [43], the assembly of specialized chromatin domains in *T. brucei* and a possible connection with RNAi remains shrouded in mystery.

Thirdly, we identified 7 large inverted repeats in the *T. brucei* genome and all generated small RNAs, albeit to different levels. In itself these long inverted repeats are a curiosity, since in many organisms such structures can have a serious effect on genome stability [44]. In particular, the remarkably high sequence identity suggests some selective pressure maybe relying on the formation of a hairpin structure at the DNA or RNA level. It is tempting to speculate that RNAi might play a role in maintaining these repeats.

Fourthly, our data highlighted two annotated pseudogenes that are a source of small RNAs, whereas the majority of pseudogenes, i.e. VSGs and ESAGs, do not generate small RNAs in procyclic trypanosomes. In addition, *Tb*DCL2, and not *Tb*DCL1, appeared to be mainly responsible for these pseudogene-derived small RNAs. Our results seem to be in contrast with a recent study in bloodstream-form trypanosomes, where small RNAs originating mainly from VSG and RHS pseudogenes were found to be dependent on *Tb*DCL1 [45]. However, since we have noted differences in the contribution of the two dicers in the generation of small RNAs from inverted repeats (Table 3), one cannot exclude a similar scenario for pseudogene-derived small RNAs, especially considering that the two studies were done in different life-cycle stages.

Lastly, we detected siRNAs at all convergent transcription units, although the distribution of the small RNAs varied greatly. At present we can only speculate about the functional significance for the existence of siRNAs originating from CTUs. Our analysis of steady-state mRNA levels at two selected CTUs (Figure 6) in wild-type and DCL2KO cells would indicate that RNAi does not play a role in the modulation of mRNA levels at these loci in procyclic cells. However, it is possible that the number of siRNAs originating from CTUs, namely 8,088 and 7,394 for the two we tested, is too low to have a direct effect on gene expression. Alternatively, siRNAs from CTUs could induce DNA or chromatin modifications at the homologous genomic locus. Another open question is the origin of these siRNAs, i.e. how is the dsRNA generated in the absence of evidence that the converging transcription units overlap. At present it is not known how and where transcription terminates at CTUs, although the presence of siRNAs at CTUs might suggest that transcription proceeds to some extent into the adjacent gene cluster. In support of this hypothesis our recent transcriptome studies using RNA-Seq [46] revealed a low level of anti-sense transcription at CTUs (unpublished observation), providing a possible avenue for the formation of dsRNA. Experiments are ongoing to corroborate this scenario and to investigate the uneven distribution of small RNAs among CTUs.

## Conclusions

The results presented here significantly expanded our earlier sequencing and functional studies that retroposon- and CIR147 repeat-derived siRNAs represent the major sources of small RNAs and expanded the repertoire to include small RNAs originating from inverted repeats, pseudogenes and loci of converging transcription units. At the same time, our data set derived from procyclic form trypanosomes did not reveal the presence of miRNAs, as well as tRNA- or snoRNA-derived RNA fragments generated by the RNAi pathway. However, this conclusion does not rule out the possibility that other stages of the trypanosome life cycle might generate a different set of small RNAs.

Our data revealed a dominant role for the nuclear *Tb*DCL2 in the endogenous *T. brucei* RNAi pathway and the landscape of siRNAs in this ancient eukaryotic parasite closely mirrors that described in metazoans, such as Drosophila and mouse, attesting to the early evolutionary origin of RNAi.

## Methods

### Standard methods

Previously published procedures were followed for culturing trypanosome YTat 1.1 cells [15], generation of knockout cell lines by PCR-based methods [47] and Northern blots of total RNA [21]. Oligonucleotides used to prepare probes for Northern blots are listed in Additional file 5.

### Small RNA library preparation, sequencing and read processing

TAP-tagged *Tb*AGO1 was expressed in wild-type cells [23], *Tb*DCL1KO and *Tb*DCL2KO cells (this study) and following *Tb*AGO1 affinity purification, the bound small RNAs were prepared for sequencing. For the library construction we essentially followed the protocol suggested by the manufacturer (http://www.illumina.com/).

Libraries were sequenced on an Illumina GAII platform at the Yale Center for Genome Analysis and the reads of 35 nt in length were pre-processed using the FASTX-toolkit on the public Galaxy webserver ([48-50]; http://galaxyproject.org/). Following removal of the adaptor sequences, reads were collapsed to non-redundant datasets and short reads (<= 17 bp) and low complexity reads (including poly A/C/G/or T, di-nucleotide repeats, etc.) were removed. We mapped processed reads, both total and non-redundant reads, to the *T. brucei* 11 mega chromosomes (GeneDB version 5) using Bowtie [51] and the Lasergene 9.1 software package from DNASTAR (http://www.dnastar.com/).

## Competing interests

The authors declare that they have no competing interests.

## Authors’ contributions

CT participated in the design of the study, analyzed the data and drafted the manuscript. HS performed the molecular studies. JBF participated in the bioinformatics analysis. EU conceived the study, participated in its design and coordination and edited the manuscript. All authors read and approved the final manuscript.

## Supplementary Material

Additional files 1 Summary of total and unique reads. Click here for file

Additional files 2 **Total and unique siRNAs at two pseudogenes in wild-type (wt),*dcl1***^***−/−***^** and *dcl2***^***−/−***^** cells.** siRNAs were normalized to the total number of reads (setlength). Click here for file

Additional files 3 **Total small RNAs at each convergent transcription units (CTUs) in wild-type (wt), *dcl1***^***−/−***^** and *dcl2***^***−/−***^** cells.** siRNAs were normalized to the total number of reads (setlength). Click here for file

Additional files 4 Sequences of oligonucleotides used for preparing probes for hybridization. Click here for file

Additional files 5 **Total small RNAs at various regions of the genome with no clear features.** Listed as "miscalleneous" in Table 2. Click here for file
