# Supplementary material for: Small interfering RNA-producing loci in the ancient parasitic eukaryote Trypanosoma brucei
Source: BMC Genomics. 2012 Aug 27;13:427. doi: 10.1186/1471-2164-13-427 (PMC3447711; doi:10.1186/1471-2164-13-427)
Supplement: Additional files 4 — Sequences of oligonucleotides used for preparing probes for hybridization. [file 1471-2164-13-427-S4.docx]

**Additional File 5:** Sequences of oligonucleotides used for preparing probes for hybridization.

*Figure 4:* IR3-specific probes (1:1 mixture of the following two oligonucleotides):

5’-TTACAGACATCGTTTCCGAGGATCA-3’

5’- TTACTGATCGGCATTCTGTTGGAGG-3’

*Figure 5:* CTU50-specific probes (1:1 mixture of the following two oligonucleotides):

5’-AAGCAACTTAAAAAGAACTAAGAGC-3’

5’-TTCTTCGTCAGATCGATTGCAGGAC-3’

*Figure 6:* CTU-specific probes (PCR fragments generated with the following two oligonucleotides):

Tb927.10.2390

5’-ACTGATGTCTCGCTTCGACCCTTAAC

5’-CCAACAAACTTGCTTGCAGTCGGTC-3’

Tb927.10.2410

5’-ACCCAACTGTTAGGAACCGCTATC-3’

5’-CAGTATCCAGCGTATGAGGATGG-3’

Tb11.01.1140

5’-GAGAATGTGGTTATGGAAGCGGG-3’

5’-GTTTGCACGAACGAATGTATGGAAC-3’

Tb11.01.1170

5’-CAACAGCAAAACCGCACAGAAATTG-3’

5’-CTCTCCTCTTTAGCAGAGAGAGGCC-3’

Loading controls:

*5S rRNA* (PCR fragment generated with the following two oligonucleotides):

5’-ACGACCATACTTGGCCGAATGC-3’

5’-ACAACACCCCGGGTTCCAGC-3’

*tRNA*:

5’-GTTGGTTTCGATCCAACG-3’
